# Supplementary material for: Antenatal depression: Associations with birth and neonatal outcomes among women attending maternity care in Harare, Zimbabwe
Source: PLoS One. 2023 Jul 7;18(7):e0270873. doi: 10.1371/journal.pone.0270873 (PMC10328234; doi:10.1371/journal.pone.0270873)
Supplement: Attachment — Submitted filename: Responses_to_Reviewers_Jan2022..docx [file pone.0270873.s001.docx]

17 January 2022

The Editor

PLoS One

**RE. Responses to reviewer**

Thank you very much for reviewing our manuscript. The comments and suggested by the reviewers have been helpful. Please find below our responses to their comments.

**Reviewer #1**: This is a very interesting paper aiming to examine the prevalence of antenatal depression and its associations with birth and neonatal outcomes in Zimbabwe. The manuscript is clear, well written and discusses about an important topic. However, there are several pieces of information missing and several points that require improvement, as described below:

**Response:** Thank you very much for this observation. We have attempted to add the missing information as well as address your comments.

**Comment 1:** Abstract:

Please make it already very clear in the abstract what are the main aims of the study.

*Thank you very much for this observation. Please note that we have clarified this, and the abstract’s introduction now reads: “Antenatal depression is highly prevalent and is associated with negative birth and neonatal outcomes. However, the mechanisms and causality behind these associations remain poorly understood as they are varied. Given the variability in whether associations are present, there is need to have context-specific data to understand the complex causal factors that go into these associations. This study aimed to assess the associations between antenatal depression and birth and neonatal outcomes among women attending maternity care in Harare, Zimbabwe.”*

**Comment 2:** Introduction

**Comment 2a:** Throughout the text, there are some typos and grammar mistakes. Please proofread and review the paper carefully.

*Thank you for this observation. Please note that we have proofread the manuscript.*

**Comment 2b:** The introduction is very well-written, but I am missing a more thorough review of the studies conducted in Africa, but specifically in Zimbabwe.

*Thank you for this comment. Please note that to date, there are no studies in Zimbabwe that assessed the associations between antenatal depression and birth/neonatal outcomes in spite high prevalence of antenatal depression (M. Kaiyo-Utete & T. Magwali, 2020; Nyamukoho et al., 2019)*.

**Comment 2c:** Also, some citations are missing (e.g. “Its under treatment has been associated with negative effects on the mother and her developing foetus.“;“ paucity of evidence on the associations of antenatal depression in the Zimbabwean context, which has a high prevalence of antenatal depression“). The reader has no clue if certain statements refer to the African or to the global context or if they are based on assumptions or evidence. Please cite accordingly.

*Thank you for this observation. Please note that the references have been added.*

*“Its undertreatment has been associated with negative effects on the mother and her developing foetus (Mitchell & Goodman, 2018).”*

*“paucity of evidence on the associations of antenatal depression in the Zimbabwean context, which has a high prevalence of antenatal depression (M. Kaiyo-Utete & T. Magwali, 2020; Nyamukoho et al., 2019),”*

**Comment 3:** Methods

**Comment 3a:** Was the validation of the PHQ-9 and EPDS done with this same population or just in the same health centres?

*Thank you for this comment. The validation of the PHQ-9 and EPDS was done with this same population. We used the baseline data to validate the screening tools.*

**Comment 3b:** Please describe in detail how the randomization process for the maternity clinics was conducted.

*Thank you for this comment. We have described the randomization process for the maternity clinics; please see in text.*

*“There are 12 polyclinics under the Harare City Council Directorate. The researcher wrote each clinic’s name on a piece of paper and placed the 12 pieces of paper in a basket. She asked a colleague to randomly pick two pieces of paper out of the basket. The clinics picked were thus selected as study sites for data collection”.*

**Comment 3c:** Did the participants provide written consent? If yes, please clearly state it.

*The participants provided a written informed consent. We have stated this in the manuscript.“Written informed consent was obtained from the prospective participants. Pregnant women aged ≤16 years were considered emancipated adults; therefore, no parental consent was obtained.”*

**Comment 3d:** Please describe in details the power analysis conducted to decide on the number of participants needed.

*Thank you for this observation. The sample size was calculated based on the validation phase. The main aim of the study was to validate depression screening tools and the assessment of the association between antenatal depression and birth/neonatal outcomes was secondary.*

**Comment 3e:** The prenatal depression diagnostics was done with the SCID interview. How did you account for physical symptoms relevant to the depression diagnosis which are normal in pregnancy?

*Thank you very much for this enquiry. During data collection, the study psychiatrists who were administering the SCID-IV had to probe about the participant’s symptoms. For example, if the stated that they were experiencing changes in appetite, sleep and concentration, the psychiatrist further asked when these symptoms started and if the patient felt they were directly caused by the pregnancy. Of note was the sleeping pattern, the psychiatrist asked questions like: “Do you feel you cannot sleep properly because you cannot find a comfortable sleeping position?”*

**Comment 3f:** What is the relevance of the variable “initiated breastfeed within 1h post-delivery” for the research question? How is this associated with prenatal depression given that many times, depending on the maternity care centre procedures, this initiation or lack of, is not a maternal choice?

*Thank you for this comment. We included the variable “initiated breastfeeding within 1hr post-delivery” because, according to World Health Organization, new mothers are encouraged to initiate breastfeeding within one hour post-delivery to guarantee that the new-born infant receives colostrum which contains antibodies that protect the infant from infections. We appreciate that in most cases this is not a maternal choice, but it has been found that depressed pregnant women are less likely to initiate breastfeeding early (Abdul Raheem et al., 2019; Eastwood et al., 2017; Figueiredo et al., 2014)or to exclusively breastfeed (Cato et al., 2019; Dias & Figueiredo, 2015). These factors have a bearing in the growth and development of the baby*.

**Comment 3g:** Did the authors account for current pregnancy complications or only for past complications? If not current, why not?

*We did not account for current pregnancy complications since we did not collect this data. Pregnant women who presented at the polyclinics and had current complications were referred to a tertiary hospital for further management. The women will therefore not be seen again at these clinics.*

**Comment 3h:** Please cite the studies supporting the choice for the confounding variables. I am unsure if they belong to the global or regional level.

*Thank you. Please see in text the references.*

**Comment 3i:** Authors mentioned that participants were recorded as depressed or non-depressed. But from those scored as depressed, what is the percentage/proportion of minor and major depression? Please add this to the text.

*Thank you for this comment. We have added this in text.*

**Comment 3j:** Authors mentioned that the depressed participants were referred to the mental health nurse or psychotherapy. When did participants started with this treatment? What kind of treatment was given? How has this impacted mental health measurements postpartum? You need to adjust for this important variable in the analysis.

*Thank you for this observation. When the study psychiatrist made a diagnosis of depression after administering the SCID-IV, the participant was given a referral letter to the mental health nurse of for psychotherapy. The research assistants directed the participants to the nurse in charge at the clinic who would then take the participant for treatment as suggested by the study psychiatrist. Unfortunately, we did not follow up to assess whether the participant received the recommended treatment. We do appreciate this in the discussion and state it as one of our study limitations.*

*Although the pregnant women who had SCID-diagnosed minor/major depression were referred to the Friendship Bench or mental health nurse for further management, information on management outcomes was not available; the fact that women may have received treatment for antenatal depression could explain some of the lack of association between antenatal depression and many birth and neonatal outcomes.*

**Comment 4:** Results

**Comment 4a:** Were there any differences between the participants who remained until the end of the study and the N=21 who were lost in the follow up?

*There were no differences between those that remained until the end of the study and those lost to follow up.*

**Comment 4b:** Participants can be either primipara or multiparas, not both. Please correct it in the text.

*Thank you for this observation. This has been corrected.*

**Comment 4c:** Table1: the authors mentioned that covariates were chosen based on previous literature and evidence, but aren’t religion and social support quite important in the regional context?

*Thank you for this observation. The covariates were chosen based on previous literature and evidence. Religion and social support are important factors associated with antenatal depression (Kaiyo-Utete et al, 2020; Nyamukoho et al, 2019); however, there is paucity of evidence that supports their effects on birth and neonatal outcomes, hence they were not included among the covariates.*

Also, were the randomly selected clinics settled in a rural or urban region? This should be acknowledged, given that if they are in an urban setting, women attending it might not be comparable to those in a rural setting with less resources to attend and pay for the maternity care.

*Thank you for this observation. The clinics are in urban areas. We have highlighted this in the methodology section.*

**Comment 4d:** Please state the percentage of participants diagnosed with minor and major depression in the sample in Table 1.

*Thank you for this observation. 29/84 (34.5%) of the depressed had major depression compared to 55/84 (65.5%) who had major depression.*

**Comment 4e:** Although the authors clearly described depression levels and infant characteristics in the postpartum phase, it would be interesting to see a table with the outcomes from babies whose mothers were antenatally depressed. It is unclear, especially considering prenatally depressed mothers were offered and potentially received treatment during pregnancy, how many of those were still depressed in the postpartum and the potential effects/improvement on baby outcomes for those receiving therapy.

*Thank you for this comment. Please not that the depression levels described in this manuscript are for pregnant women. The outcomes, though, include for both depressed and non-depressed pregnant women to assess associations with antenatal depression. Depression in pregnancy was assessed using the SCID-IV while depressive symptoms in the postpartum period were assessed using the EPDS at 6 weeks postnatal clinic visit.*

**Comment 4f:** Table 2: It would be important to add the percentage of babies not being breastfed at all (e.g. only formula feeding).

*Thank you for this comment. Formula feeding is not very common in the sample due to the financial costs attached to formula feeding hence the reason we categorised it as non-exclusive breastfeeding.*

**Comment 5:** Discussion

**Comment 5a:** In the second paragraph, authors justify the association between antenatal depression and LBW risk behaviours. However, it is important to contextualize it to your sample. Based on Table 1, the participants have a low percentage of risk behaviours. Maybe you could justify it if the depressed moms have a higher rate of risky behaviours upon comparison with the non-depressed one. But this needs to be described in text or tables.

*In our study, there was no significant association between birth weight and LBW; this could because of the bias introduced in the study due to face-to-face interviews which might have influenced the participants to give socially desirable answers.*

**Comment 5b:** Authors also mentioned cultural barriers associated with breastfeeding. What are those in Zimbabwe? It would be important to contextualize it in the region where the study was conducted as well as with the sample here studied.

*Thank you very much for this comment. Please see in the text, we have added references that contextualize the cultural barriers associated with breastfeeding in Zimbabwe (Muchacha & Mtetwa, 2015; Mundagowa et al., 2019; Nduna T, 2015; Yaya et al., 2020).*

**Comment 5c:** Please make sure to make it clear that the association between prenatal depression and exclusive breast was quite small and how relevant is it.

*Thank you for this comment. Please note that we have addressed it in the text.*

**Comment 5d:** Did the authors considered that the lack of statistical significance between depression and certain outcomes may be due to the fact that most participants were diagnosed with minor depression? Since this is not clearly described in text, the reader has no clue if this is a possibility. Please make it clear.

*Thank you for this comment. We have added the proportions of minor and major depression among the participants and this consideration.*

**Reviewer #2:**

In this original article, the Authors aim to determine the association between antenatal depression during the second or third trimester and birth and neonatal outcomes assessed at 6 weeks post-partum in a cohort of 354 Zimbabwean women. They found that a diagnosis of major and minor antenatal depression was associated with low birth weight in the new-borns, with a lower rate of exclusive breastfeeding and with higher rate of depressive symptoms beyond the EPDS cut-off.
The study is interesting because to date only few prospective studies evaluated the impact of prenatal depression in sub-Saharian countries. Some methodological issues, hovewer, lower my enthusiasm for the manuscript. Specifically the authors considered the antenatal depression as the main predictor of ten different outcomes. In a second step of the analyses they controlled for eight different covariates. Moreover they dichotomised most of the variable with the risk of losing some important information.
If possible I’d like the authors to address the following queries:

**Introduction**
• The authors should consider adding a reference to the sentence “Its undertreatment has been associated with negative effects on the mother and her developing foetus” (see Mitchell & Goodman, Arch Womens Ment Health, 2018)

*Thank you for this. We have added the reference as per your suggestion.*

• When referring to the studies that failed in identifying an association between depressive symptoms and neonatal outcomes the authors might consider to discuss the role of symptoms severity, comorbid anxiety and medications (see Ossola et al., J Matern Fetal Neonatal Med, 2021). This seems particularly relevant as it is not clear whether (or in which percentage) the sample was treated.

*Thank you. We have noted that in our discussion.*

• When stating the aims the authors conclude that “if antenatal depression is associated with poor birth and neonatal outcomes, it would emphasize the importance of screening for it as part of routine prenatal care”. This however is only partly descriptive of the study design as the authors also consider post-partum depressive symptoms as a possible maternal outcome. This should be clarified early on.

*Thank you. We have clarified. It now reads:* “*If antenatal depression is associated with poor birth and neonatal outcomes and postnatal depressive symptoms, it would emphasize the importance of screening for it as part of routine prenatal care.”*

**Methods and Results**

• I’m not sure I fully understand the meaning of “a medical doctor attending to severe chronic conditions monthly”. Do the authors mean that an obstetrician-gynaecologists was available only once a month for the most severe patients? Was the psychiatrist that administered the SCID-IV similarly available?

*Thank you for this comment.*

*The clinics do not have a resident medical practitioner at all; they are staffed by midwives and general nurses. In cases of obstetric complications, pregnant women are referred to tertiary hospital for further management. However, once a month, a medical practitioner (not a specialist) visits the clinic to attend to patients with chronic illnesses such as hypertension, diabetes mellitus and heart conditions. The psychiatrists who administered the SCID were part of the research team, they were not stationed at these clinics.*

• The authors detailed the power analysis as follow “Assuming a 39% prevalence of antenatal depression, we calculated that a sample of 366 women was needed to achieve a 5% precision at 95% confidence intervals”. I’m not sure I understand based on which aim this sample size was calculated.

*Thank you for this observation. The sample size was calculated based on the validation phase. The main aim of the study was to validate depression screening tools and the assessment of the association between antenatal depression and birth/neonatal outcomes was secondary.*

• When defining the data collection procedures would be helpful to detail which information were collected at baseline (see Table 1) and how these were dichotomised. As noted above the authors should justify why they dichotomised the following variables: maternal age, maternal BMI, birth weight at delivery, gestational age, Apgar score, postnatal EPDS. Whereas this is useful to represent the data, doing so they are likely to lose nearly a third of the information (see Altman & Royston, BMJ 2006).

*Thank you for this comment. We dichotomised the variables to make it easier to represent the data. However, when we ran the point-biserial correlation with these variables as continuous variables there were no differences in the associations, hence we did not change the analysis in the manuscript.*

• Out of the n=84 women, how many women were depressed in the second and in the third trimester?

*Thank you. We did not analyse depression status per trimester because the women were recruited either in the second trimester or third trimester.*

• Would also be interesting to know how many women were diagnosed with minor and major depression as these have different risk factors (see Marchesi, Bertoni & Maggini, Obstet Gynecol, 2009).

Did the authors found the same results when splitting the predictor in these two groups?

*Thank you for this observation.* *29/84 (34.5%) of the depressed had major depression compared to 55/84 (65.5%) who had major depression. For this paper, we did not split the data into minor and major depression. The study was more of an exploratory study to assess whether there are any associations between antenatal depression and birth/neonatal outcomes.*

• When defining the confounding variables is not clear how the authors selected eight variables out of the seventeen available as covariates. A reference would be needed here. Especially considering that they excluded alcohol use from the covariates but in the discussion, they state that “increased risk behaviours such as alcohol use and smoking which may ultimately affect the foetal intrauterine growth”. Which of the available variables in Table 1 were associated with the selected outcomes in Table 2?

*Thank you for this comment. Please see in text; we have added the references on the chosen covariates.*

• Following the previous comment, did the authors explore the possible interactions between the predictors? For example, does the alcohol mediate the association between antenatal depression and low birth weight? Does any of the known risk/protective factors associated with post-natal depression interact in the association between antenatal depression and the EPDS score?

*There were no significant relationships between predictors as we tested for collineality thus we ruled out multicollineality.*

• It’s not clear to me why they used point-biserial correlations “to measure the strength and direction of the association between antenatal depression and continuous outcomes such as infant’s weight and height at six weeks postdelivery” but not with gestational age and weight at birth. Would also be interesting at least to know whether they found an association between antenatal depression and birth weight and gestational age as continuous variables. Also what’s the difference between point-biserial correlations coefficients and the beta in a linear regression?

*Thank you for this comment. We ran the point-biserial correlations to measure the strength and direction between antenatal depression and*

a. *Gestational age at birth (*r*_pb_=0.024, p=0.65)*
b. *Birthweight (*r*_pb_=0.096, p=0.07)*
c. *Apgar score at 5 minutes (*r*_pb_=0.049, p=0.361)*

*Just like with the logistic regression, the point-biserial correlation showed that there was a borderline association between antenatal depression and birthweight as a continuous variable but there were associations between gestational age and Apgar score.*

*The point-biserial correlation was used we were assessing associations between a binary variable (antenatal depression) and a continuous variable (infant’s weight and height at 6 weeks postdelivery) whereas a linear regression assesses associations between continuous variables.*

• Including so many predictors as in the multivariate logistic regression might results in multi-collinearity issues. Did the authors test for the association among the predictors? Especially when dichotomising the covariates it is possible that some subgroups have in fact zero subjects (e.g., in a 2 by 2 table how many women have chronic illnesses diagnosed during the pregnancy but no chronic illnesses before the pregnancy?). I’m asking because this might affect the p-value.

*We tested pairwise relationship and they were not correlated. There were no groups with zero subjects. But the changes in the p-values are purely due to additive effects.*
• I think I don’t understand the meaning of the sentence “The low uptake of exclusive breastfeeding could be due to cultural barriers that young mothers face when trying to implement this”. Could the authors pleas spell out this part of the discussion?

*The sentence “The low uptake of exclusive breastfeeding could be due to cultural barriers that young mothers face when trying to implement this” means that not many mothers are exclusively breastfeeding their babies. The reasons for this could be due to cultural barriers, such as the young mother being encouraged to supplement the baby’s feeding by the elderly women in the family. We have spelled this out in the discussion.*

**Minor points**

• In table 4 the asterisks are missing for the association between HIV status and assisted delivery and infant illness at 6 weeks. Also “predictable” might be changed into “predictors”.

*Thank you for this observation. Please note that we have put the asterisks in Table 4.*

• I’m not a native English speaker but I spotted a couple of typos (e.g. “Maternal postnatal depressive symptoms at six weeks postdelivery post-delivery” and “recorded in the baby’s medical booklet Research team members checked”). Also the last paragraph of the “Data collection procedure” seems redundant with the methods section. The authors should probably proofread the manuscript.

*Thank you so much for this comment. We have attended to the typos.*

Once again, we thank you for reviewing our manuscript.

Kind regards

**Malinda Kaiyo-Utete**

On and behalf of the authors
